# Clinical and neuroimaging correlates of antiphospholipid antibodies in multiple sclerosis: a preliminary study

**DOI:** 10.1186/1471-2377-7-36

**Published:** 2007-10-18

**Authors:** Carlos J Bidot, Lawrence L Horstman, Wenche Jy, Joaquin J Jimenez, Carlos Bidot, Yeon S Ahn, J Steven Alexander, Eduardo Gonzalez-Toledo, Roger E Kelley, Alireza Minagar

**Affiliations:** 1 Wallace H. Coulter Platelet Laboratory, University of Miami Dept. of Medicine, Miller School Of Medicine, Miami, FL, USA; 2 Departments of Molecular and Cellular Physiology, Louisiana State University Health Sciences Center, Shreveport, LA, USA; 3 Department of Radiology, Louisiana State University Health Sciences Center, Shreveport, LA, USA; 4 Department of Neurology, Louisiana State University Health Sciences Center, Shreveport, LA, USA

## Abstract

**Background:**

The presence of antiphospholipid antibodies (APLA) in multiple sclerosis (MS) patients has been reported frequently but no clear relationship between APLA and the clinical and neuroimaging features of MS have heretofore been shown. We assessed the clinical and neuroimaging features of MS patients with plasma APLA.

**Methods:**

A consecutive cohort of 24 subjects with relapsing-remitting (RR) MS were studied of whom 7 were in remission (Rem) and 17 in exacerbation (Exc). All subjects were examined and underwent MRI of brain. Patients' plasma was tested by standard ELISA for the presence of both IgM and IgG antibodies using a panel of 6 targets: cardiolipin (CL), β2 glycoprotein I (β2GPI), Factor VII/VIIa (FVIIa), phosphatidylcholine (PC), phosphatidylserine (PS) and phosphatidylethanolamine (PE).

**Results:**

In exacerbation up to 80% of MS subjects had elevated titers of IgM antibodies directed against the above antigens. However, in remission, less than half of MS patients had elevated titers of IgM antibodies against one or more of the above antigens. This difference was significant, p < 0.01, for all 6 target antigens. Interestingly, none of the MS patients had elevated plasma titers of IgG against any of the target antigens tested. Correlation analysis between MRI enhancing lesions and plasma levels of APLA revealed high correlation for aPC, aPS and aFVIIa (p ≤ 0.0065), a trend for aPE and aCL (p = 0.056), and no correlation for aβ2GP1. The strongest correlation was for aFVIIa, p = 0.0002.

**Conclusion:**

The findings of this preliminary study show that increased APLA IgM is associated with exacerbations of MS. Currently, the significance of this association in pathogenesis of MS remains unknown. However, systematic longitudinal studies to measure APLA in larger cohorts of patients with relapsing-remitting MS, particularly before and after treatment with immunomodulatory agents, are needed to confirm these preliminary findings.

## Background

Multiple sclerosis (MS) is an immune-mediated neurodegenerative disorder of human central nervous system, which is initially characterized by loss of myelin/oligodendrocyte complex followed by progressive neuronal loss and axonal degeneration [1-3]. Clinically, the majority of MS patients present with a relapsing-remitting course and within a few years, a large number of these patients with or without treatment with immunomodulatory agents enter another phase of disease known as secondary progressive MS. Neuropathologically, MS lesions within MS are characterized by perivenular infiltration of myelin basic protein-activated CD4 T lymphocytes as well as reactive macrophages which orchestrate the massive inflammatory cascade within the CNS [2]. Another arm of the immune system, the humoral immune system-autoantibodies as well as activated complement system-also play a significant role in the pathogenesis of MS [2,3]. The abnormal activation of both cellular and humoral immune arms combined with disruption of the blood brain barrier (BBB), activation of the cerebral endothelial cells, and loss of adjacent tight and adherent endothelial junctions [4-6] precede formation of perivenular demyelinating lesions

The first antiphospholipid antibody (APLA) identified was anti-cardiolipin (aCL) in 1941, seen in false-positive syphilis tests. The lupus anticoagulant (LAC), which is believed to be a manifestation of APLA, was originally associated with a hemorrhagic diathesis [7,8] but in the 1980s, a stronger association with thrombosis was found, first called aCL syndrome or Hughes syndrome, now known as antiphospholipid syndrome (APS) [9-12]. A major advance was the realization that nearly all APLA are in fact directed not against phospholipids (PL) *per se*, but against PL-binding proteins [13]. The first such cofactor identified was β2GPI, said to be associated with thrombosis, but many others were subsequently identified, now numbering in the dozens. This discovery has greatly broadened the definition of APLA, and makes clear that APLA detected by standard ELISA methods are in reality very heterogeneous [14,15].

High frequencies of APLA are seen in autoimmune disorders other than systemic lupus erythematosus (SLE), not necessarily associated with thrombosis, such as in the bleeding disorder, immune thrombocytopenic purpura (ITP) [16] and in MS.

The neuropsychiatric manifestations of APLA (with or without APS) are well known [17,18] and in some instances resemble those of MS [19-23]. The reported frequencies of positive APLA in MS have ranged from 10% or less [24-26] to 44% [27] and to 88% [20]. Such wide discrepancies are common in the APLA literature and are attributable to variations in methodological details (which are inadequately specified in some of the above cited reports) as well as criteria of patient selection such as distinguishing clinical state. To our knowledge, no report to date has established a clear association between APLA and the clinical state or radiologic imaging data in MS patients. The present study was motivated to clarify these uncertainties using the same standardized methods we have applied in several other studies [16,28,29] and a well-defined patient population.

## Methods

### Patient population

The study was approved by the Institutional Review Board of Louisiana State University Health Sciences Center in Shreveport, Louisiana and all subjects provided their signed informed consent forms. We measured plasma APLA in a cohort of 24 subjects with relapsing-remitting MS (RRMS) defined by the revised McDonald criteria [30], of whom 17 were in exacerbation (Ex) and 7 in remission (Re). None of the study subjects had any other medical disorders or causes (other autoimmune disorders, drug abuse, taking certain medications such as antibiotics, hydralazine, procainamide or other inflammatory disorders) that could explain positivity for APLA. None of the female study subjects had any history of spontaneous abortions or stroke-like syndromes. Exacerbation was defined as the appearance, the reappearance or the worsening of symptoms of neurological dysfunction lasting more than 24 hours preceded by at least one month of stability from the last relapse. All subjects were recently diagnosed and treatment-naive. In parallel, we also measured APLA in 21 healthy subjects (controls) with no prior history of immune-mediated disorders.

#### Imaging methods

Brain MRI was performed using a 1.5 T machine with a standard quadrature head coil. The image protocol included sagittal T1-, axial T1-, T2-weighted, and fluid attenuated inversion recovery (FLAIR) images. Sagittal T1-Weighted: 550/10 ST:5 mm, Acq matrix:256 × 192; Axial T1-weighted 700/10 ST: 5 mm Acq matrix 256 × 192; Axial T2GRE> 550/15 ST:5 mm; Dual echo (T2WI/PD). 3000/140/14 ST:5 mm Acq matrix 256 × 224. All MRI images of the brain were obtained pre- and post-intravenous infusion of single dose of Gd-DTPA. We performed qualitative analysis of axial T2-weighted and pre- and post-contrast axial T1-weighted images for evaluation of MS lesions. The MR images were independently analyzed by a neuroradiologist blinded to patients' clinical data. Identification of MS lesions was made by visual inspection as was determination of the enhancing versus non-enhancing lesions. Computer based software allowed comparison of the lesions among different groups. Subjects were subgrouped into those with versus without axial T1-weighted at least one contrast enhancing lesions in order to determine any correlation between the presence of APLA and the presence of contrast-enhancing lesions.

### Measurement of APLA

#### 1. Sample handling

Blood was collected in blue-top Vacutainers (citrate), centrifuged at 4500 rpm for 15 min, and plasma was frozen at -20 until assay. We use plasma not serum to avoid potential loss of some antibodies in the clot. Upon defrosting, samples were again centrifuged and 1:50 dilutions were made in PBS-Tween as described [28].

#### 2. Materials

Phospholipids were obtained from Sigma Chemicals (St. Louis, MO). Purified β2GPI and factor VII were obtained from Enzyme Research Laboratories (South Bend, IN), or activated recombinant factor VII (rFVIIa, NovoSeven) from NovoNordisk, A/S (Bagsvaer, Denmark). Commercial kits for assay of aCL and β2GPI were obtained from INOVA Diagnostics, San Diego, CA (Cat. No's. 708625, 708630, 708665, 708670 for aCL IgG, IgM and aβ2GPI IgG, IgM, respectively).

#### 3. Target antigens

IgG and IgM APLA were assayed by ELISA for β2GP-I and factor VII/VIIa (FVII/VIIa), a novel APLA antigen [28]. In addition, reactivities against four pure phospholipids (PL) were measured: cardiolipin (CL), phosphatidylcholine (PC), phosphatidylserine (PS), and phosphatidylethalonamine (PE).

#### 4. ELISA methodology

Guidelines of the European Consensus [31,32] were observed in development of the aCL assay, and similar methodology for the other tests, as detailed previously [28,29]. Our methodology was validated with commercial kits approved by the Federal Drug Administration (FDA) for aCL and aβ2GPI, identified above. The kits included calibrator mAb and gave good agreement between our results and known positive and negative samples furnished with the kits. Since the particular protein target antigens (or cofactors) in plasma are unknown for the four PL tested (CL, PC, PS PE), we estimated titers by the OD ratio method described below.

#### 5. Normal control values and cutoff points

To have a constant reference for calculating optical density (OD) ratio, pooled plasma from 21 normal volunteers was used, termed the quality control (QC) sample. The OD of this QC plasma was used as the denominator for calculating the OD ratio: OD of patient sample/OD of QC plasma. To determine normal ranges, we tested 50 healthy subjects (32 female, 18 male) of age range 25–60 years, by the same OD ratio method and set the positive cutoff at 2 SD above the mean. Only 1 of 50 normal controls was found outside this range (IgG for β2GPI and CL). Results did not differ substantially if the cutoff was set at 3 SD. Each plate was run with one QC plasma sample and usually with an additional individual normal control, all in duplicate.

#### Statistical analyses

All statistical analyses were performed using StatMost 32 software. Comparisons for significant differences were determined by chi squared test. Correlations were evaluated in terms of the Pearson correlation coefficient. Significance was accepted if p < 0.05. In case of multiple analyses (6 APLA tests), then the Bonferroni adjustment may apply. The standard of significance changes from 0.05 to 0.0083 (= 0.05/6) for each test.

## Results

Demographic features of MS subjects are presented in Table 1. The study subjects with RRMS consisted of 20 females and 4 males with a mean duration of neurological symptoms prior to diagnosis of 5.3 months. None of the MS subjects had ever experienced any thromboembolic or thrombo-occlusive syndromes.

**Table 1:** Demographic features of MS subjects

	Exacerbation	Remission
No	17	7
Age	23 ± 4	24 ± 3
Female/Male	13/4	6/1
Mean EDSS	3.5	3.0
MRI enhancing lesions		
Present	12	0
Absent	5	7

Race (Caucasian) N = 24

**Figure 1:**
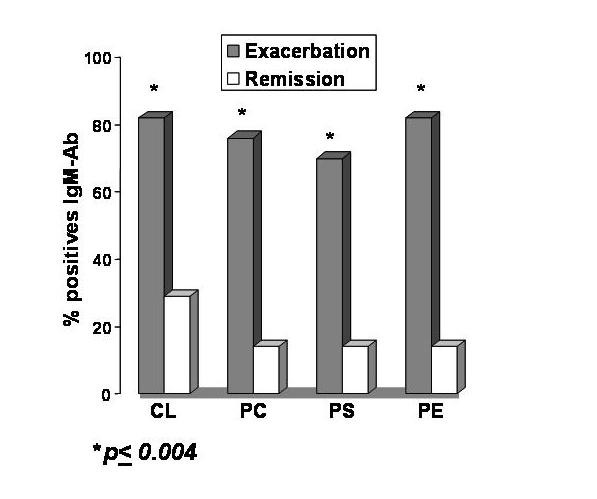
Comparison of positive IgM antibodies (IgM-Ab) in MS subjects, for phospholipids antigens, in exacerbation versus remission. Abbreviations: CL = cardiolipin, PC = phosphatidylcholine, PS = phosphatidylserine, PE = phosphatidylethanolamine.

The principal finding of this study was a much higher frequency of APLA positive test results for MS patients in exacerbation compared to remission. Figure 1 shows graphically the results for subjects with IgM antibodies against the pure phospholipids (PL). It is observed that the increased frequency of seropositivity ranged from 2-fold to 4-fold in exacerbation compared to remission. This was statistically significant in every case at p < 0.004. Interestingly, no positive IgG was encountered.

It is apparent that if all patients were combined rather than separated by exacerbation vs. remission, the frequency of positive tests would be reduced. For example, on the basis of all 24 patients, the prevalence of aPS was 54% and that of aFVII was 41.6% (not shown).

**Figure 2:**
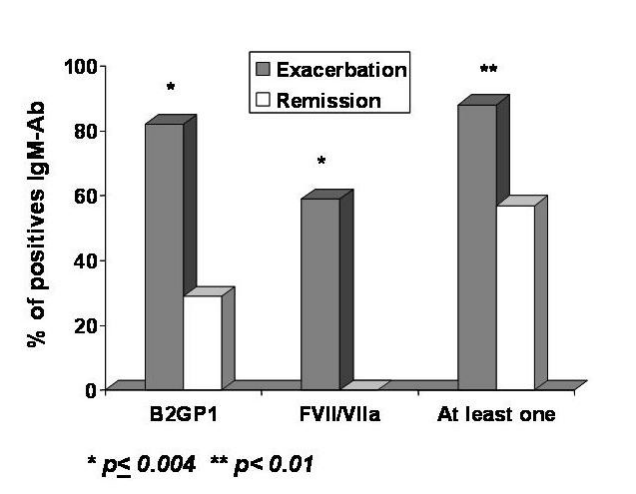
Comparison of positive APLA-IgM antibodies (IgM-Ab) in MS subjects, for β_2 _glycoprotein-I (B2GP-I), factor VII/VIIa (FVII/VIIa) and for at least one of the 6 measured antigens, in exacerbation versus remission.

Figure 2 shows positive results for the two protein antigens, β2GPI and FVII/VIIa. Both of these autoantibodies were significantly and more frequently present in exacerbation compared to remission, p = 0.004. Remarkably, none of the patients in remission exhibited seropositivity for anti-FVII but 10 of 17 (59%) in exacerbation were positive.

A summary of frequency of seropositivity of APLA in MS patients and controls is shown in Table 2. Plasma of 19 of our MS subjects tested positive in at least one of the 12 tests (6 antigens, IgG and IgM). All these antibodies were of IgM family. No positive IgG was detected. The right-most bar of Figure 2 shows frequency of at least one positive of the 12 tests between exacerbation and remission. This difference was also significant (p < 0.01).

**Table 2:** Frequency of positivity of APLA in MS and controls

**Target antigens**	**Exacerbation**		**Remission**		**Controls**	
	
	**IGG**	**IGM**	**IGG**	**IGM**	**IGG**	**IGM**
β2-GP-I	0/17 (0%)	14/17 (82%)	0/7 (0%)	2/7 (28%)	1/21 (4%)	0/21 (0%)
VIIa	0/17 (0%)	10/17 (59%)	0/17 (0%)	0/7 (0%)	0/21 (0%)	0/21 (0%)
CL	0/17 (0%)	14/17 (82%)	0/17 (0%)	2/7 (28%)	0/21 (0%)	0/21 (0%)
PC	0/17 (0%)	13/17 (76%)	0/17 (0%)	1/7 (14%)	0/21 (0%)	0/21 (0%)
PS	0/17 (0%)	12/17 (71%)	0/17 (0%)	1/7 (14%)	0/21 (0%)	0/21 (0%)
PE	0/17 (0%)	14/17 (82%)	0/17 (0%)	2/7 (28%)	0/21 (0%)	0/21 (0%)

**At least one**	**0/17 (0%)**	**15/17 (88%)**	**0/17 (0%)**	**4/7 (57%)**	**1/21 (4%)**	**0/21 (0%)**

The abbreviations: β2-GP-I = β2-glycoprotein-I; VIIa: Factor VIIa; CL = cardiolipin; PC = phosphatidylcholine; PS= phosphatidylserine; PE = phosphatidylethanolamine

Next, we analyzed possible correlations between the presence of T1-weighted post contrast enhancing lesions on brain MRI and the presence of various sub-classes of APLA. Results are shown on Table 3. A significant correlation between contrast-enhancing lesions and 3 of the 6 target antigens (FVII, PC, PS) was found (p < 0.0002, 0.0065, 0.0014, respectively). There was a trend toward correlation for anti-PE and anti-CL (p = 0.056 for both). Interestingly, there was no trend of correlation for anti-β2GPI.

**Table 3:** Correlations of APLA with MRI enhancing lesions

	Pearson	
APLA	Corr. Coeff.	p value
FVII IgM	0.686	0.0002
PC IgM	0.529	0.0065
PS IgM	0.602	0.0014
PE IgM	0.387	0.056
CL IgM	0.380	0.056
β2GP-I IgM	0.220	0.29

After converting patients' positivity and negativity data of APLA and MRI lesions into numeric "1" and "0", respectively, Pearson correlation analysis was performed.

Abbreviations: VIIa = Factor VIIa; PC = phosphatidylchlorine; PS = phosphatidylserine; PE = phosphatidylethanolamine; CL = cardiolipin; β2-GP-I = β2-glycoprotein-I

## Discussion

The central finding of this paper is a close association between APLA and certain clinical and neuroradiologic features of MS. We surmise that previous studies failed to detect this association because they did not distinguish clinical states of the patients, or used methods unable to make this discrimination.

The frequency of MS plasma positive for APLA in this study was higher than in most but not all previous reports, since as noted in the introduction, Ijdo and colleagues found 88% positivity [20]. The number of tests performed on each specimen in our study was larger than most and would help to increase the likelihood of finding at least one positive test in a given subject. Furthermore, few if any prior studies clearly separated exacerbation from remission, resulting in a reduced apparent prevalence, since we clearly show a much greater frequency of positivity in exacerbation.

Our finding of IgM exclusively (no IgG) was also unexpected but is not entirely inconsistent with prior reports since most find a preponderance of IgM over IgG. For example, Sugiyama et al found 14 of 32 positive for IgM but only 3 of 32 positive for IgG [27]. It is unlikely that our finding of exclusively IgM reflects a systematic methodological error since other studies in progress at the same time (such as of ITP) revealed nearly equal numbers of IgG and IgM seropositivity. Additionally, another recent study on clinical and neuroimaging correlates of autoreactive antibodies in MS patients revealed similar finding [33]. Through a retrospective study, the investigators determined that APLA mainly of IgM type was present in 55% of their study subjects with MS. It remains unknown now why, in our MS subjects, exclusively IgM APLA was found; however, we hypothesize that since the subjects in this study were all newly diagnosed they had not yet class-switched to IgG. Alternatively, one may suggest that a class-switching occurs in a small fraction of APLA+ MS patients, but majority of these APLA+ MS patients are defective in class-switching [34].

The clinical significance of the findings of this study is unknown; however, there are two broad possibilities: first, that APLA in MS may contribute causally to the disease pathogenesis, or that APLA are secondary (epiphenomenal) with no role in promotion of the inflammatory cascade of MS. Regarding the first concept, it is important to point out that our assay of aPC, aPS, aPE and aCL does not identify the specific plasma antigen responsible for a positive test. As remarked in the introduction, many APLA antigens are now known, and certainly many more exist, therefore it is possible that APLA in MS are directed against some specific antigen that could be involved, for example, in compromise of the BBB. A number of available reports document cross-reaction of some APLA with endothelial cells and platelets [35-39], raising the possibility that the rise of specific types of APLA in the MS subjects could trigger exacerbations by further compromise of the BBB. We have shown complex effects of plasma from MS patients on brain microvascular endothelial cells in tissue culture [40,41].

It should be stressed that our assay against the four pure phospholipids will detect any PL-binding protein, not necessarily those thus far identified as APLA. It is therefore possible that we are detecting an antigen specifically involved in compromise of the BBB, possibly responsible for the endothelial activation that we reported in exacerbations of MS [42].

It is of interest that only anti-β2GPI, which is believed to be a risk factor for thrombosis, was not associated with MS radiologic imaging, while anti-FVII was most closely associated (Table 2). The pathophysiological significance of these findings remain unclear but it may be relevant to note that FVIIa is closely associated with tissue factor (TF), FXa, and TF pathway inhibitor (TFPI) in normal hemostasis, and that TF has numerous actions apart from thrombosis and hemostasis [43].

In light of these findings, further investigation of APLA in a larger cohort of MS subjects is warranted to clarify the significance of these autoantibodies in MS. Further understanding the role of these autoantibodies in MS subjects may eventually translate into more effective treatments.

## Conclusion

The findings of this preliminary study demonstrate presence of IgM APLA during exacerbations of MS. Currently, the significance of these autoantibodies in pathogenesis of MS remains unknown. Longitudinal studies to measure both IgM and IgG classes of APLA in larger cohorts of patients with relapsing-remitting MS are necessary to establish the significance of these autoantibodies and assess their pattern of expression before and after treatment with disease modifying agents.

## Competing interests

Dr. Minagar has received an independent medical grant from EMDSerono, Inc. All other authors have nothing to disclose.

## Authors' contributions

CJB, LLH, and CB Jr provided laboratory expertise to measure plasma APLA, obtained the data, and prepared the manuscript.

WJ, JJJ, YSA, JSA, AM, and REK contributed to the manuscript by designing the study, recruiting the study subjects, interpreting clinical and laboratory data, performing statistical analysis, and preparing the manuscript.

EGT contributed to the manuscript by interpreting the neuro-radiology data, capturing data, and preparing the manuscript.

All authors have read and approved the contents of the final manuscript.

## Pre-publication history

The pre-publication history for this paper can be accessed here:


